# Science is not a Social Opinion

**DOI:** 10.3390/dj7020034

**Published:** 2019-04-01

**Authors:** Marco Tatullo

**Affiliations:** 1 Tecnologica Research Institute, 88900 Crotone, Italy; marco.tatullo@tecnologicasrl.com; Tel.: +39-3498742445; 2 Department of therapeutic dentistry; IM Sechenov First Moscow State Medical University, Moscow 119146, Russia

**Keywords:** evidence-based medicine, science dissemination, big data in dentistry

## Abstract

Recently, the main American associations in the dental field reported concerns regarding a film on the Netflix, Amazon, Apple, and Vimeo platforms that reported that endodontic treatments of root canals are linked to serious systemic pathologies, against any scientific evidence. This extreme case highlights how information, in a social networking era, is dramatically conditioned by a small number of users, leading to large scale consequences in political opinions, alimentary choices, or even in healthcare policy. It is urgent to demonstrate a strong awareness by the academic, institutional, and associative bodies in order to restore the correct flow of information on mass media and social networks.

“Without data you’re just a person with an opinion.” This is one of the most reported phrases of W. Edwards Deming, who correctly described that science does not require opinions, but evidence-based facts. However, we are even more used to reading about false information, widespread as a scientific truth. Grinberg et al. reported that the proliferation of fake news during the 2016 election cycle on Twitter was extremely concentrated, involving only 1% of individuals for about 80% of fake news source exposures [1]. This analysis highlights how information, in a social networking era, is dramatically conditioned by a small number of users, leading to large scale consequences in political opinions, alimentary choices, or even in healthcare policy. 

Akhi et al. recently discovered a significant association between salivary IgA-to-malondialdehyde acetaldehyde-modified low-density-lipoprotein and atherosclerosis, describing as pathognomonic the presence of specific oral pathogens in patients affected by coronary artery disease [2]. The crosslink between oral and systemic conditions has been widely and seriously investigated in several evidence-based articles, however, we are increasingly used to reading false information on common dental pathologies related to severe systemic diseases, which have been widespread as scientific truth. 

Recently, the main American associations in the dental field reported concerns about a film on the Netflix, Amazon, Apple, and Vimeo platforms, which reported that endodontic treatments of root canals were linked to serious systemic pathologies, against any scientific evidence. Unfortunately, despite being well established that the oral environment can be influenced by several systemic diseases, however, several mass media have still disregarded the severe implications of some shocking unreliable messages about specific dental treatments [3]. False information often creates fake myths and could be dangerous for public health [4]. Finally, fake news and false science have led Italian scientists to conduct several public information campaigns aimed at limiting the drastic reduction in the percentage of vaccinations in newborns [5]. This trend shows how fake news today has an impact on the vast majority of users in several social networks, therefore, it is urgent to demonstrate a strong awareness by the academic, institutional, and associative bodies in order to restore the correct flow of information on mass media and social networks. Mister Deming will thank us.
